# Predictive Value of Serum VEGF Levels in Non-Small Cell Lung Cancer: A Review

**DOI:** 10.32604/or.2025.066228

**Published:** 2025-09-26

**Authors:** Eleni Kokkotou, Andriani Charpidou, Nikolaos Syrigos

**Affiliations:** Oncology Unit, Third Department of Medicine, “Sotiria” General Hospital for Chest Diseases, National and Kapodistrian University of Athens, Athens, 11527, Greece

**Keywords:** Non-small cell lung cancer (NSCLC), vascular endothelial growth factor (VEGF), VEGF receptors (VEGFRs), biomarker, tumor angiogenesis

## Abstract

Vascular endothelial growth factor (VEGF) and its receptors (VEGFRs) serve an essential role in tumor angiogenesis and have emerged as potential therapeutic targets in lung cancer. This review explores the significance of serum VEGF levels as a predictive biomarker in non-small cell lung cancer (NSCLC). The VEGF family, consisting of VEGFA, VEGFB, VEGFC, VEGFD, and placenta growth factor (PlGF), engages with specific receptors, including tyrosine kinase receptors (VEGFR-1, VEGFR-2, and VEGFR-3) and neuropilin receptors (NRP-1 and NRP-2), to promote angiogenesis and lymphangiogenesis. VEGF-A, the primary component of the VEGF family, binds to VEGFR-2 to stimulate endothelial cell proliferation and migration, while VEGF-B, VEGF-C, and VEGF-D interact with VEGFR-1 and VEGFR-3 to regulate tumor angiogenesis, lymphangiogenesis, and metastasis. The VEGF/VEGFR signaling pathway activates various downstream effectors, including phospholipase Cγ1, mitogen-activated protein kinase (MAPK), and phosphatidylinositol 3-kinase (PI3K)/protein kinase B (Akt), which are essential for maintaining vascular homeostasis and promoting angiogenesis. In NSCLC, elevated serum VEGF levels have been observed, and the VEGF/VEGFR axis is frequently impaired, leading to irregular blood vessel formation and metastatic spread. Despite the development of anti-VEGF therapies, their impact on lung cancer outcomes has been limited. Further research is needed to optimize the effectiveness of these treatments and elucidate the potential of serum VEGF as a predictive biomarker in NSCLC.

## 1 Introduction

Lung cancer is a significant contributor to cancer-related mortality in humans. Due to its hostile nature, it is more often identified at a late stage and has an unfavorable prognosis. There are two main categories of lung cancer, non-small cell lung cancer (NSCLC) and small-cell lung cancer (SCLC) [1]. NSCLC corresponds to around 80%–85% of cases, while SCLC, a more aggressive tumor, accounts for approximately 15% [2]. Research on lung cancer has resulted in understanding biology and along with the use of predictive biomarkers and treatment advancement, has led to improved patient outcomes. Additionally, public health actions at reducing prevalence of tobacco use have resulted in lower incidence of lung cancer and better survival rates in high-income countries. The frequency of lung cancer incidence is decreasing more rapidly in men than in women, which reflects the historical patterns of tobacco use and cessation between genders. On the contrary, new lung cancer cases are still rising in low-income countries due to lack of public health smoking cessation measures and due to limited access to healthcare. Moreover, lung cancer can still be diagnosed in patients who have never smoked [2].

Bevacizumab, a humanized monoclonal antibody, is the first anti-angiogenic drug since 2006 that was used in combination with chemotherapy as first-line treatment for NSCLC. By binding to vascular endothelial growth factor-A (VEGF-A), it obstructs its linkage with vascular endothelial growth factor receptor (VEGFR). VEGFR thus inhibits the stimulation of VEGF signaling pathways that lead to neovascularization [3]. *In vivo* studies found that bevacizumab impedes angiogenesis, facilitates the regression of neovascularization, and normalizes the vasculature to enable the delivery of cytotoxic chemotherapy, and it also has a direct effect on cancer cells [4]. According to its mode of action, the clinical development of bevacizumab was focused on tumor types characterized by angiogenesis. The introduction of bevacizumab to standard chemotherapy treatment offered an unusual and efficient treatment option for advanced cancers with poor prognosis in the era before the development of targeted therapy and immunotherapy. Survival rates of lung cancer have been increased due to systematic therapy, with the combination of programmed cell death protein 1 (PD-1)/programmed death-ligand 1 (PD-L1) antibodies with platinum-doublet chemotherapy as the current standard of care for first-line treatment of advanced non-small-cell lung cancer (NSCLC) without a known targetable mutation, regardless of PD-L1 score. Driver mutations like EGFR, KRAS, BRAF, and ALK were also detected, resulting in the development of targeted drugs [2]. However, these medicines only provide advantages to certain groups of patients with specific molecular changes, and resistance mechanisms often restrict their effectiveness. This emphasizes the necessity for a more profound comprehension of the development of lung cancer and the discovery of novel targets for treatment [5].

Tumor angiogenesis, which involves the factors and signaling pathways, has emerged as a promising focus for therapeutic interventions in different malignancies, including lung cancer. VEGF and its receptors (VEGFRs) stimulate endothelial cell growth, movement, and infiltration via angiogenesis. VEGF promotes vascular permeability, aids in creating a temporary structure for the movement of endothelial cells and boosts the attraction of vascular precursor cells from the bone marrow. Recent research indicates that VEGF targets tumor cells explicitly, hence promoting the growth and spread of cancer. Both non-small cell lung cancer (NSCLC) and small cell lung cancer (SCLC) patients have been found to have increased expression of VEGF in the bloodstream [4]. Despite multiple anti-VEGF medications that are now in clinical development or have already been approved, their impact on lung cancer outcomes has been limited, with only minor improvements observed compared to the encouraging preclinical results [5,6].

## 2 Prospective Targeting of VEGF in Lung Cancer

### 2.1 Overview of VEGF/VEGFR Axis

**Figure 1:**
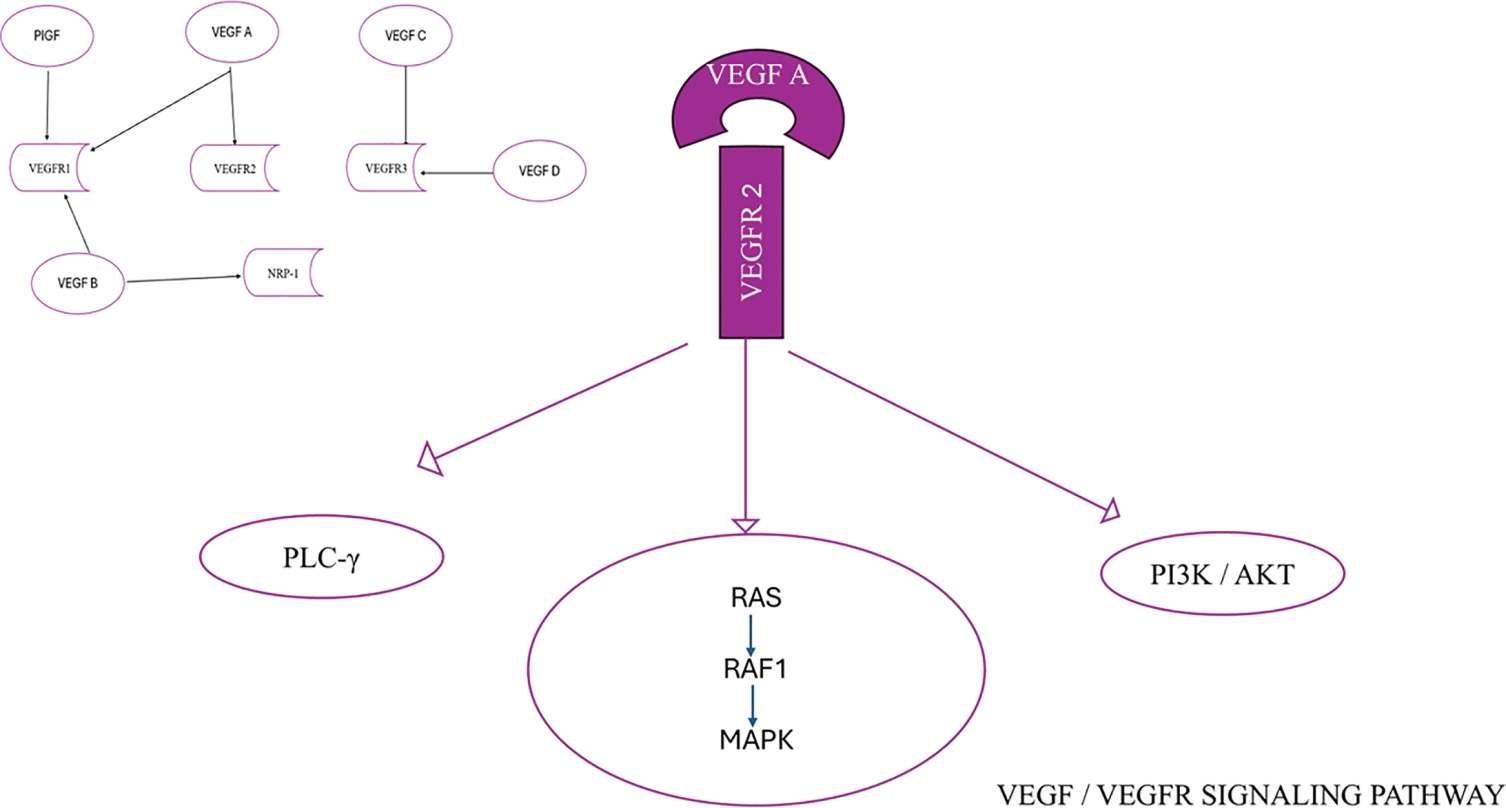
VEGF/VEGFR signaling pathway

The VEGF family, consisting of VEGFA, VEGFB, VEGFC, VEGFD, and placenta growth factor (PlGF), plays an integral part in the mechanism of angiogenesis. The functions of the members of the VEGF family are processed by the process of connecting to their particular receptors. VEGF receptors are classified into two types: tyrosine kinase receptors (VEGF receptors, VEGFR), consisting of VEGFR-1, VEGFR-2, and VEGFR-3, and neuropilin receptors (NRPs), which include NRP-1 and NRP-2 [7,8]. NRPs function as co-receptors for VEGF, and the connection between VEGF and NRPs enhances the stability of the receptor complex [7]. The members of the VEGF family exhibit a specific affinity for VEGFR. VEGF-A is the primary constituent of the VEGF family that promotes the formation of new blood vessels. It is present in all vascular tissues, macrophages, tumor cells, and other types of cells [9,10]. Furthermore, it can attach to both VEGFR-1 and VEGFR-2. Still, it mainly attaches to the latter to form a pair, self-phosphorylates, and activates, thereby playing a vital role in subsequent signaling processes. This leads to the growth and movement of endothelial cells and carries out duties related to forming new blood vessels [11,12]. VEGF-B mainly attaches to VEGFR-1 and NRP-1 and significantly impacts tumor angiogenesis and the enhancement of ischemic damage situations [13,14]. VEGF-C and VEGF-D mostly attach to VEGFR-3 and contribute to the process of lymphangiogenesis [14,15]. VEGF-D is linked to the spread of tumors to nearby lymph nodes [16,17]. Additionally, PIGF primarily attaches to VEGFR-1 and controls the development and maturity of blood arteries by preventing the multiplication of endothelial and parietal cells [18] (Fig. 1).

VEGFR-1 is expressed in numerous cell types other than endothelial cells and has an important function in regulating leukocyte migration. VEGFRs consist of seven immunoglobulin (Ig) homology regions, which contain the area where the ligand binds. Additionally, they have an intracellular domain that exhibits tyrosine kinase activity, which is responsible for transmitting signals within the cell. VEGF contact induces the activation of phospholipase Cγ1, the mitogen-activated protein kinase (MAPK) pathway via Ras/Raf1 activation, and the phosphatidylinositol 3-kinase (PI3K)/protein kinase B (Akt) pathway (Fig. 1). Phospholipase Cγ1 holds a critical role in controlling the concentration of Ca^2+^ ions within cells and the production of endothelial nitric oxide synthase. The collective impact of these series of events is crucial for preserving the fundamental stability of blood vessels and facilitating processes such as the formation of new blood vessels, cell division, and migration of cells [19,20]. The VEGF/VEGFR signaling system is frequently altered in several cancer types, which results in the formation of malformed blood vessels and the spreading of metastatic cancer cells [19–21].

### 2.2 Role of VEGF in Angiogenesis and Cancer

**Figure 2:**
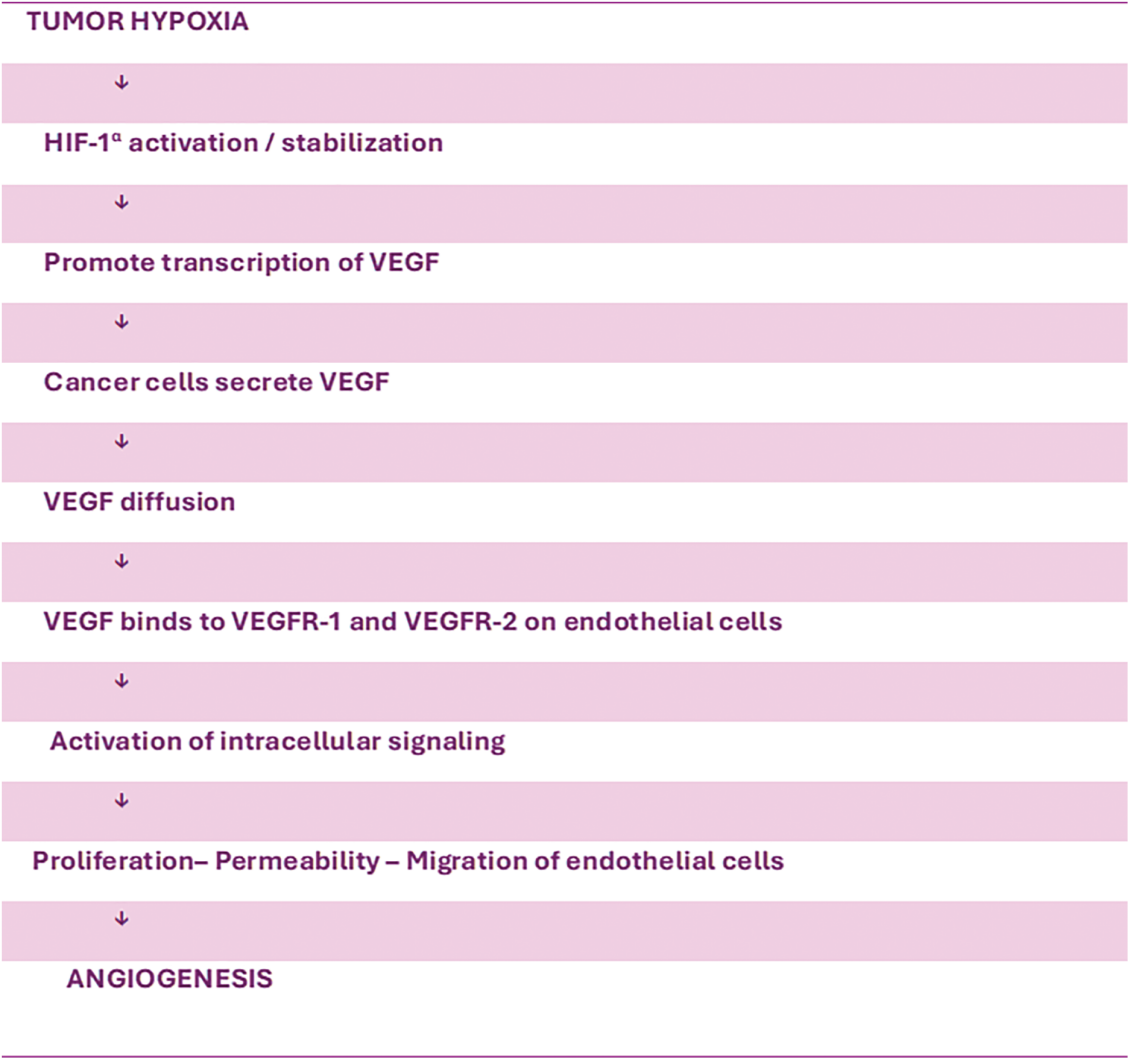
Tumor hypoxia and angiogenesis

Several proteins and genes that play a crucial role in controlling the cell cycle, angiogenesis, and apoptosis have been identified as markers that greatly influence the response to treatment and clinical prognosis in patients with non-small cell lung cancer (NSCLC) [22,23]. Angiogenesis is a vital factor for the proliferation and metastasis of tumors and has been found to be a separate prognostic factor [24]. VEGF is a crucial and significant activator of tumor angiogenesis. Among the conflicting influences of proangiogenic and antiangiogenic factors, a signal appears that stimulates the generation of VEGF. This specific signaling system enables VEGF to perform many functions in the process of neoangiogenesis [21]. VEGF expression mostly appears in endothelial cells, but it has also been detected at high levels in several types of tumors, including lung tumor cells. Hypoxia produces increased levels of VEGF in tumor tissue and stabilizes and enhances the expression of the transcription factor Hypoxia-inducible factor-1α (HIF-1α). HIF-1α, in turn, promotes the transcription of VEGF, which is then released, diffuses through the tissue, and binds to specific receptors on the surface of endothelial cells [25] (Fig. 2). Several studies indicate a correlation between the genetic variation of VEGF and the sensitivity, prognosis, and therapeutic responsiveness of individuals with NSCLC [26].

### 2.3 Role of VEGF in the Procedures of Tumor Microenvironment (TME) Cell Components in NSCLC

According to studies, VEGF plays a role in malignancies by inducing the growth of new blood vessels (angiogenesis), but also by affecting tumor cells [27]. VEGF may stimulate the formation and spread of tumors by binding to receptors found on tumor cells through autocrine and paracrine processes [28]. NRPs, together with tyrosine kinases, have the ability to control the activity and motion of growth factor receptors and integrins. This makes them essential in aiding the effects of VEGF on malignant cells [28]. Malignant cells evade the immune response by inhibiting the function of T cells, such as by elevating the levels of T cell checkpoints [29,30]. VEGF-A promotes the production of PD-1 and other suppressive checkpoints, like cytotoxic T-Lymphocyte-associated protein 4 (CTLA-4), on the surface of T cells. Moreover, it impedes the operation of CD8^+^ T cells, resulting in a persistent malfunction that ultimately hinders the effector role of T cells [31,32]. Recent research indicates that tumor hypoxia, angiogenesis, and immunosuppression could mutually disrupt each other, fostering tumor progression and reducing the efficacy of cancer therapy [33]. VEGF not only directly modulates T cell activity but also potentially suppresses T cell function by regulating the levels of Fas ligand (FasL). VEGF-A amplifies the presence of FasL in the TME [34,35]. FasL is present in the outer layer of T cells and in cancer endothelial cells, while being absent in a healthy vascular system. The presence of FasL in endothelial cells in human carcinomas leads to the reduction of CD8^+^ T lymphocytes [26,36]. Regulatory T cells (Tregs), often called Treg cells, are an essential group of CD4^+^ T cells. Various preclinical and clinical studies have shown that Treg cells are a predominant kind of immunosuppressive cell observed in malignancies [26]. They inhibit the process of immune surveillance to counteract cancer in individuals with favorable medical conditions. They impede the ability of patients with tumors to develop anti-tumor solid immunity, which leads to the formation and advancement of different types of malignant tumors, such as NSCLC [37]. The expression of VEGF-A in cancer patients was found to relate strongly with the levels of intratumoral regulatory T cells (Tregs) [38]. VEGF-A can promote the development of Tregs by increasing the population of immature dendritic cells (DCs) [39]. In addition, VEGF-A can directly control the recruitment of Treg cells in the TME by binding to VEGFR2. This interaction boosts the proliferation of Treg cells and enhances their immunosuppressive activity [26,39,40]. Tumor-associated macrophages (TAMs) are versatile cells that can adopt various polarization states. They play a crucial role in the initiation and advancement of cancer [41]. TAMs are found at every stage of tumor formation, making them the most prevalent immune cells in the TME [42]. There are two distinct phenotypes of TAMs, namely M1 and M2. The M1 phenotype has tumor-suppressing actions, while the M2 phenotype facilitates tumor advancement [43]. TAMs produce cytokines, chemokines and growth factors that induce immunosuppression and activate the suppressive immunological checkpoint proteins in T cells [44]. Hwang et al. [45] demonstrated that M2 TAMs significantly increased VEGF-A and VEGF-C expression levels in non-small cell lung cancer (NSCLC) cells. On the other hand, M1 TAMs only increased the expression levels of VEGF-A in NSCLC cells. This indicates that TAMs play a significant role in the development of blood vessels and lymphatic vessel formation, promoting the advancement of NSCLC [45].

A type of cell called dendritic cells has the highest potential to present antigens compared to other cells. They can produce cytokines and facilitate the development of effector T and Natural Killer (NK) cells [46,47]. DCs can be separated from the first phase of hematopoietic progenitor cells (HPC), and VEGF-A may contribute to this mechanism by binding to HPC CD34^+^ cells through VEGFR-1 and thus suppressing the activity of nuclear factor-κB (NF-κB), which activates transcription factors in these cells. As a result, the differentiation and maturation of DC are inhibited [48,49]. VEGF can potentially hinder the function of dendritic cells by increasing the expression of PD-1. Blocking the development of dendritic cells decreases the infiltration of T cells into tumors and has an immunosuppressive impact [50]. Recent data reveal that VEGF might impair mature DCs’ migratory ability and immunological activity through the VEGFR-2-mediated RhoA-cofilin1 pathway [50].

Elevated levels of immature DCs in cancer patients are correlated with heightened levels of VEGF, which play a role in facilitating the malfunction of DCs [48]. In addition, the findings of a clinical trial examining the connection between DC infiltration and VEGF expression in NSCLC (132 primary NSCLC patients who underwent surgery) revealed that the average number of infiltrating DCs in the group with high VEGF expression was lower than that in the group with low expression [51]. This suggests that VEGF might control the infiltration of DCs into NSCLC tumors.

VEGF-A is a factor that can enhance the proliferation of myeloid-derived suppressor cells (MDSCs). The MDSC population comprises diverse and varied immature myeloid cells, which serve as progenitor cells for macrophages, DCs, or granulocytes [52]. MDSCs are defined by their origin in the bone marrow, immature state, and ability to suppress the immune response [52]. These factors can enhance the survival of tumor cells, stimulate the growth of new blood vessels (angiogenesis), facilitate the invasion of tumor cells, and accelerate the spread of cancer to other parts of the body (metastases) [52,53]. In addition, MDSCs can promote immunological tolerance and decrease the activity of effector T cells and NK cells, hence stimulating immune responses. Furthermore, MDSCs can hinder the proliferation of T cells specific to tumors and facilitate the formation of regulatory T cells (Tregs), which are crucial in suppressing the immune response and evading the immune system. MDSCs are also implicated in the process of Treg cell development. An elevation of myeloid-derived suppressor cells (MDSC) in the bloodstream of individuals with cancer leads to a reduction in the number of fully developed dendritic cells (DCs) [54]. Many studies have indicated that MDSCs play a significant role in modulating a range of tumor-related immunosuppressive activities and tumor immune escape, including NSCLC [26].

NK cells are a specific subset of cytotoxic innate lymphoid cells within the innate immune system. They possess a distinct ability to eliminate tumor cells effectively. VEGF can impede the development of NK cells by obstructing the maturation of DCs [48,55]. In addition, VEGF can enhance the quantity of MDSCs and suppress the activity of NK cells, resulting in immunological escape [26]. Research has demonstrated that NK cells are capable of releasing VEGF-A when exposed to low oxygen circumstances, which is a distinguishing feature of the TME [56]. In settings of low oxygen levels (hypoxia), the release of VEGF is temporary. This is because when NK cells return to the bloodstream, this occurrence can be reversed. Hypoxia plays an essential part during cancer treatment by causing an imbalance in the signaling between pro- and antiangiogenic variables and physical compression. This results in abnormal blood vessels and substantially decreased blood flow in tumors. The increasing heterogeneity in blood flow, which worsens over time, differs depending on the stage and location of tumor growth. This leads to cancer cells evading the immune system, enhancing their ability to move in and spread to other body parts, and exerting selected survival pressures [57]. By relieving hypoxia, it is possible to alter the characteristics of macrophages, making them more supportive of tumor growth and suppressing the immune response, improving the efficiency of cancer treatment.

Except for hypoxia-driven resistance and tumor microenvironment modulation, there are also other mechanisms of resistance to anti-VEGF treatment in NSCLC. Tumors might express alternative pro-angiogenic factors, including fibroblast growth factors (FGFs) [58], platelet-derived growth factor (PDGF), and angiopoietins, thereby avoiding dependency on VEGF, they can promote endothelial cell proliferation, migration, and survival. Certain genetic variations in tumors may enhance resistance to anti-VEGF treatment, by making tumor cells nutrient deprived, or, alternatively, by promoting VEGF-independent tumor angiogenesis as well as other modes to recruit blood vessels [59]. For example, p53 inactivation is known to render tumor cells less sensitive to hypoxia-induced apoptosis [60]. Another mechanism is vascular mimicry, which describes the capacity of hostile tumor cells to create vessel-like networks autonomously, without involving endothelial cells or angiogenic factors. This process allows tumors to circumvent traditional angiogenesis and to maintain their nutrients and oxygen despite anti-VEGF treatment. It can be found mainly in tumors that are metastatic and are associated with a poor prognosis. During this process, tumor cells express endothelial-associated genes like VE-cadherin and MMPs that are able to form perfusable channels [61–63].

### 2.4 VEGF and Immune Checkpoints in NSCLC

VEGF not only stimulates tumor growth by facilitating the formation of new blood vessels but also affects different immune cells in the tumor microenvironment, suppressing the immune response. Thus, while treating NSCLC, choosing VEGF-VEGFR-targeted medications can impede tumor growth.

VEGF is upregulated in NSCLC, with higher expression levels observed in the tumor tissue compared to the adjacent normal lung tissue [61]. Kondo et al. [64] primarily acknowledged the potential of VEGF as a serum diagnostic marker for malignant diseases. Their research found that VEGF levels in the sera of cancer patients were substantially elevated compared to those without cancer. Many studies have followed and explored the predictive and prognostic associations of circulating VEGF in NSCLC and other cancer types. Some of them showed no association between circulating VEGF levels and chemotherapy response in NSCLC patients [65,66]. However, Lissoni et al. [67], found that higher pretreatment VEGF levels correlated with poorer chemotherapy responses in patients with NSCLC and colorectal carcinoma. Similarly, studies into the prognostic role of VEGF in chemotherapy have yielded conflicting results. Zang et al. [68] found that high VEGF levels tended to be associated with a poor prognosis, and patients with higher VEGF levels had shorter PFS vs. lower VEGF levels. Shibaki et al. [69] found that only high levels of serum VEGF were significantly associated with a shorter PFS in older patients (aged ≥75 years) and those with poor performance status (PS 2). Their results demonstrated that serum VEGF concentration may be a negative predictive biomarker in elderly and poor PS advanced NSCLC patients receiving anti-PD-1 antibody treatment. Another study investigated the prognostic and predictive value of baseline and post-treatment levels of serum VEGF-A, VEGF-B, sPD-1, and sPD-L1 of advanced NSCLC patients treated with immune checkpoint inhibitors. Higher pretreatment sPD-L1 and posttreatment VEGF-B levels were found to independently predict worse overall survival, while VEGF-A and sPD-1 failed to show a significant correlation with prognosis. None of the biomarkers was associated with treatment response [70]. These discrepancies may arise from different methodologies that were used to assess VEGF concentrations, differences in histological types, sample sizes, or treatment regimens used in the study populations. Therefore, enhancing detection techniques and classification criteria could help clarify the predictive and prognostic significance of VEGF.

The elevated VEGF expression is associated with tumor recurrence, reduced survival rate, metastasis, and mortality [61,62]. VEGF is essential for tumor progression and immunosuppression. Hence, specific medications that hinder the VEGF pathway, such as monoclonal antibodies against VEGF and tyrosine kinase inhibitors (TKIs), are employed to treat NSCLC [3].

Eastern Cooperative Oncology Group (ECOG) 4599 was a phase III trial where patients with recurrent or advanced non-squamous NSCLC, excluding those with brain metastasis, hemoptysis, and performance status >1, received carboplatin plus paclitaxel with or without bevacizumab (Table 1). Patients that were randomized to the bevacizumab arm had improved progression-free survival (PFS) with median PFS of 6.2 months relative to 4.5 months compared to those that received only chemotherapy, with corresponding response rates of 35% and 15%, respectively. The group that received chemotherapy and bevacizumab treatment had better Overall survival (OS) with a median survival of 12.3 months compared to 10.3 months [71,72]. From the adverse events of the trial, clinically significant bleeding rate was 4.4% related to 0.7% of patients who received only chemotherapy. There were also 15 deaths related to chemotherapy and bevacizumab treatment, five of which were due to pulmonary hemorrhage.

**Table 1:** Bevacizumab trials in NSCLC

	ECOG 4599	AVAIL
**Treatment arms**	Arm 1: bevacizumab + carboplatin + paclitaxel	Arm 1: cisplatin + gemcitabine + bevacizumab 7.5 mg/kg
		Arm 2: cisplatin + gemcitabine + bevacizumab 15 mg/kg
	Arm 2: carboplatin + paclitaxel	
		Arm 3: cisplatin + gemcitabine + placebo
**Primary endpoint**	OS	PFS
**Results**	Improvement in OS, PFS, ORR with bevacizumab	Improvement in PFS, ORR with bevacizumab (both doses)
		OS not significantly different

Note: NSCLC, non-small cell lung cancer; OS, overall survival; PFS, progression-free survival; ORR, objective response rate.

The Avastin in Lung (AVAIL, referring to the Avastin in Lung Cancer) trial where patients with advanced or recurrent non-squamous NSCLC (including those with malignant pleural or pericardial effusion), received bevacizumab combined with cisplatin and gemcitabine had improved ORR and PFS but they did not have any significant difference in OS maybe due to utilization of effective second-line treatment (Table 1). Adverse events above grade 3 were found significantly on the bevacizumab arm, with main events of hypertension, bleeding, and proteinuria [71].

Trial E1505, a study where bevacizumab was added to the adjuvant therapy for patients with NSCLC, failed to improve OS [73]. In this trial, grade 3–4 toxicities were more statistically significant in the group that received bevacizumab, and hypertension was most frequent. Results from another study, the Ramucirumab Evaluation in Lung Cancer with Docetaxel (REVEL) trial, that included NSCLC patients that had progressed after first line treatment with platinum-based chemotherapy and those patients that received second line treatment with docetaxel-ramucirumab had improved OS, median OS of 10.5 months for the patients that received ramucirumab compared to 9.1 months for docetaxel-placebo group [74].

LUME-Lung 1 study, a double-blinded double control randomized trial, assessed the efficacy and safety of nintendanib, an oral, triple angiokinase inhibitor of (VEGFRs), platelet-derived growth factor receptors α/β, and fibroblast growth factor receptors [75], as well as the oncogenic kinases FLT-3 and RET, and docetaxel as second-line treatment for patients with advanced NSCLC.

Patients in the nintendanib group had a median PFS of 3.4 months compared to 2.7 months of the other group. Results of the study revealed that patients with adenocarcinoma had a median OS of 10.9 months to nintendanib group compared to 7.9 months in the docetaxel alone group [76]. A non-interventional LUME-BioNIS study [77] evaluated the effectiveness and safety of nintendanib plus docetaxel in patients with advanced adenocarcinoma NSCLC patients, that had received chemotherapy and immunotherapy. Patients had median OS was 8.8 months, and the median PFS was 4.6 months.

Over the past decade, immunotherapy has been used as a treatment for NSCLC. An immunological checkpoint is a protein that can induce immunosuppression, hence modulating the immune response [78]. Monoclonal antibodies that inhibit the binding of CTLA-4 and programmed PD-1 or its ligand PD-L1 have received clinical approval [79]. PD-1 and PD-L1 are predominantly present in immune cells, specifically NK cells, DC, CD4^+^, and CD8^+^ T cells [80,81]. PD-1 binds with its ligand PD-L1 to suppress the activation and reproduction of T cells, resulting in the evasion of the immune system. Strong connections were found between the levels of PD-L1 expression and the levels of angiogenic factors, including VEGFA and HIF-1α [82]. CTLA-4 is an external protein found on the surface of cells that can regulate immunological suppression. Its primary function is to activate T cell receptors, which play a crucial role in the immune response [83].

The first-line therapy, now approved as immune checkpoint inhibitors, can be classified into three primary categories: Anti-PD-1, Anti-PD-L1, and Anti-CTLA-4. VEGF-A suppresses immune activation and promotes immunosuppression by influencing different immune cells inside the TME. Thus, the suppression of immunological escape can be achieved by decreasing the impact of VEGF, followed by the combination of immune checkpoint inhibitors for the treatment of NSCLC. Initially, anti-angiogenic medications have the ability to restore normalcy to the blood vessels within tumors, resulting in an increase of tumor immune cells, specifically tumor-infiltrating lymphocytes, in cases of NSCLC [84]. Immune checkpoint inhibitors alleviate the suppression of PD-1 and PD-L1 on T cells, and the combined impact of these two factors results in improved treatment outcomes for solid malignancies.

The IMPOWER 150 trial (A Phase III Study of Atezolizumab in Combination with Carboplatin, Paclitaxel, and Bevacizumab in Patients with Advanced Non-Squamous Non-Small Cell Lung Cancer) showed that patients who were randomized onto the Carboplatin, Paclitaxel, Bevacizumab, and Atezolizumab arm had superior PFS and OS than those in other arms [85] (Table 2). The median OS for patients who received Atezolizumab was 19.2 months compared to 14.7 months in the other arm, and the median PFS was 8.3 months compared to 6.8 months in the other arm. It is not certain if those survival results are due to the addition of atezolizumab or due to the addition of bevacizumab to the treatment. The investigators of the study decided to exclude patients with EGFR or ALK genomic alterations from the primary analysis since data showed similar OS and PFS as chemotherapy [85].

**Table 2:** Anti-VEGF and ICI trials in NSCLC

	IMPOWER 150	CONTACT 01	LEAP 008	HARMONi 2
**Combination therapies**	Carboplatin, Paclitaxel, Bevacizumab, Atezolizumab	Atezolizumab, Cabozatinib vs. Docetaxel	Lenvatinib, Pembrolizumab vs. Docetaxel	Ivonescimab vs. Pembrolizumab
**Results**	Improvement in OS, PFS	OS and PFS not significantly different	OS and PFS not significantly different	Improvement in PFS

Note: VEGF, vascular endothelial growth factor; ICI, immune checkpoint inhibitor; NSCLC, non-small cell lung cancer; OS, overall survival; PFS, progression-free survival.

Another trial, the CONTACT-01 [86], evaluated the combination of Atezolizumab plus Cabozantinib in patients with recurrent metastatic NSCLC following treatment with anti–PDL1/PD-1 immunotherapy and platinum-containing chemotherapy (Table 2). Cabozantinib is a potent inhibitor of multiple receptor tyrosine kinases, like VEGFR2, MET, and RET, which play important roles in tumor cell proliferation and neovascularization, and are involved in suppression of antitumor immune responses [87]. Results from the study showed that median OS was 10.7 months in the experimental arm compared to 10.5 months in the Docetaxel arm. Also, median PFS was 4.6 months to the Atezolizumab and Cabozantinib arm compared to 4 months to the docetaxel arm. Did not improve overall survival (OS) [86]. LEAP-008 evaluating lenvatinib with or without pembrolizumab in patients with metastatic NSCLC who progressed after receiving anti–PD-(L)1 therapy and platinum-based chemotherapy (Table 2). Lenvatinib is an oral receptor TKI with activity against VEGF receptors 1–3, fibroblast growth factor receptors (FGFR) 1–4, plate-let-derived growth factor receptor-alpha (PDGFRα), and RET and KIT proto-oncogenes [88]. The final analysis of the study found that patients on the lenvatinib and pembrolizumab arm had a median OS of 11.3 months compared to median OS of 12 months on the docetaxel arm. Also, patients treated with lenvatinib and pembrolizumab had a median PFS of 5.6 months compared to 4.2 months in the docetaxel arm. Thus, LEAP trial was another trial that did not meet its primary end points did not meet its OS and PFS primary end points [89].

HARMONi 2 trial compares ivonescimab vs. pembrolizumab treatment for PD-L1 positive patients with advanced NSCLC [90] (Table 2). Ivonescimab is a bispecific antibody against programmed cell death protein 1 and vascular endothelial growth factor. The primary end of the trial was PFS, and median PFS was longer with ivonescimab (11.1 months) vs. pembrolizumab (5.8 months).

A restricted number of studies have investigated the importance of plasma VEGF levels in predicting the therapeutic outcomes of anti-angiogenesis, together with anti-PD-L1 approaches. Tozuka et al. [91] indicated that patients who had a reduction in their post-treatment plasma VEGF-A concentrations in comparison to their pre-treatment levels exhibited a markedly extended PFS in contrast to those whose post-treatment plasma VEGF-A concentrations either increased or remained stable. Within the responder cohort, a larger fraction of patients demonstrated a persistent decline in their plasma VEGF-A levels across the treatment period.

## 3 Discussion

In order to understand tumor angiogenesis and its part in the development and treatment of NSCLC, the VEGF axis must be understood. This review tries to highlight the importance and usefulness of targeting VEGF and its related pathway in NSCLC. Tumor angiogenesis, which includes many molecular factors and signaling pathways, has evolved into an attractive field of investigation for treatment in many malignancies, including lung cancer. VEGF and its receptors (VEGFRs) have an important role in promoting endothelial cell proliferation, migration, and invasion through angiogenesis. VEGF stimulates blood vessel permeability, aiding the formation of provisional extracellular matrices that support movement of endothelial cells and enhance the attraction of vascular precursor cells from the bone marrow. Results from studies found that VEGF can act directly on tumor cells and thus contribute to tumor progression and metastasis.

There is a contribution of VEGF in tumor neovascularization as well as to the transformation of the TME that leads to immune escape. Studies revealed that VEGF-A blocks the maturation of dendritic cells, impedes the activity of T and NK cells, and enhances the growth of T-regs and MDSCs. These functions make VEGF a key link between tumor angiogenesis and immune escape. Specifically, VEGF-A participates in cancer treatment by stimulating the production of PD-1 and other suppressive checkpoints on T cell surfaces, resulting in blocking T cell effector functions. This process may lead to tumor progression and decreased efficacy of cancer therapy. Also, VEGF-A may regulate FasL levels thus affecting T cell function indirectly. TAMs, especially M2 TAMs, were found to be related to increased VEGF-A and VEGF-C expression in NSCLC cells. Differentiation and maturation of DCs may be inhibited by VEGF-A hence affecting the development of effector T and NK cells. Studies found that higher VEGF levels in cancer patients are related to increased levels of immature DCs, implying that VEGF may affect the migration of DCs into NSCLC tumors. Moreover, VEGF might inhibit NK cell development, leading to immunological escape. The presence of different pro-angiogenic factors or genetic variations may contribute to anti-VEGF treatment resistance in NSCLC.

Usage of anti-angiogenic therapies such as bevacizumab and nintedanib into standard treatment regimens has given moderate but significant improvements in PFS and OS. However, intrinsic or acquired resistance, toxicity concerns, and patient heterogeneity often reverses the benefits of the treatment. Furthermore, while numerous trials have examined combinations of VEGF/VEGFR inhibitors with chemotherapy and immune checkpoint inhibitors, results have been mixed. The IMpower150 trial showed encouraging synergy between VEGF blockade and immunotherapy, but other studies, such as CONTACT-01 and LEAP-008, failed to demonstrate consistent survival benefits.

The results of the studies revealed that there is a need for validated biomarkers that can predict the patients who will benefit from anti-VEGF treatment or combination treatments. Levels of VEGF-A in the serum of the patients with NSCLC have shown prognostic relevance in some studies, but its usage is limited by conflicting findings and methodological variability. Second, tumor heterogeneity and adaptive resistance mechanisms—such as upregulation of alternative pro-angiogenic pathways (e.g., FGFs, PDGF) or vascular mimicry—further complicate treatment efficacy. Finally, the precise temporal sequencing and optimal combination of anti-VEGF therapy with immunotherapy remain unresolved.

Future research directions should concentrate on patient stratification through molecular and immune profiling, dynamic biomarker monitoring, and the investigation of rational combination strategies. Combining angiogenesis inhibitors with agents targeting other pathways (such as MET, AXL, or epigenetic regulators), or using them in neoadjuvant or maintenance settings, could provide additional benefits. Additionally, addressing the immunosuppressive effects of VEGF signaling by normalizing vessels and modulating the TME may enhance responses to immune checkpoint inhibitors.

In conclusion, while VEGF remains a validated target in NSCLC, its therapeutic exploitation requires a more nuanced understanding of tumor biology, careful patient selection, and well-designed combination strategies. Ongoing translational research and biomarker-driven clinical trials will be essential to fully harness the potential of anti-angiogenic therapy in the era of precision oncology.

## Data Availability

Not applicable.
